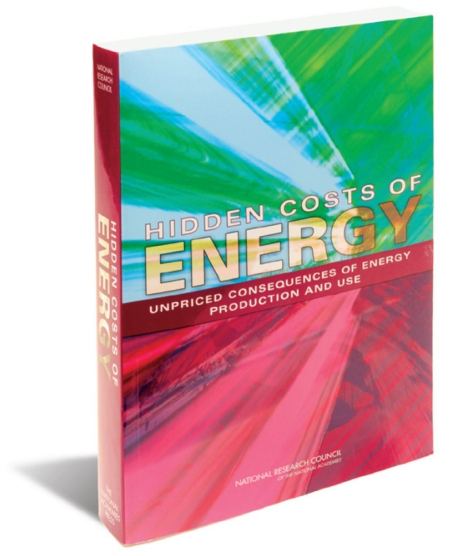# Hidden Costs of Energy: Unpriced Consequences of Energy Production and Use

**Published:** 2011-03

**Authors:** Maximilian Auffhammer

**Affiliations:** Maximilian Auffhammer is an associate professor of Agricultural and Resource Economics & International Area Studies at the University of California, Berkeley. He is an associate director of the UC Institute for Energy and Environmental Economics and a Faculty Research Fellow at the National Bureau of Economic Research. His publications focus on the impacts of climate change, the effectiveness of environmental and energy policies, and on forecasts of greenhouse gas emissions.

The production and consumption of energy and its derived services have raised the living standards of billions. Energy is traded in markets, and its price in most cases does not reflect the full cost of its use to society. Absent regulation, only the private cost of producing, for example, a kilowatt hour (kWh) of electricity is paid for by consumers through their electricity bills. These private costs, however, fail to account for a variety of external costs from this production activity in the form of, for example, present-day health damages from increased air pollution or potentially lower grain yields due to global warming halfway around the world and almost a century into the future. Economists argue that regulators should make producers “internalize” the external costs, preferably through flexible market mechanisms such as pollution taxes or cap-and-trade systems. Although qualitatively this can be easily explained to an undergraduate student in a 45-minute lecture, the exact magnitude of the full external costs is impossible to determine. The difficulty in determining the damages is caused by the significant degree of uncertainty in our understanding of the source-to-dose relationship combined with an even more limited understanding of the dose–response relationships for the vast variety of pollutants and potentially affected populations and systems. Monetizing the damages (and, in rare cases, benefits) is further complicated by the fact that many affected “receptors” of the pollution are not traded in markets and therefore do not carry a price.

The National Academy of Sciences was charged by Congress to “define and evaluate the health, environmental, security, and infrastructural external costs and benefits that are not or may not be fully incorporated into the market price of energy [or] into the federal tax or fee.” The National Research Council committee, convened for this purpose and chaired by Jared Cohon and vice-chaired by Maureen Cropper, produced the report *Hidden Costs of Energy*, which provides quantitative, qualitative, and in some cases monetized estimates of these external damages from the use of energy for electricity, transportation, heat, and infrastructure and security.

The central estimate of damages, which has been widely reported in the press, of US$120 billion for the last year of available data (2005) must be interpreted as a strict lower bound of the true external costs. Fifty-two percent of the estimated damages arises from health-related damages from the combustion of coal for electricity; 47% stems from the combustion of liquid fuels for transport. The remaining US$2 billion in damages are attributable to health effects from natural gas for electricity production and heating. To put this in perspective, the average price per kilowatt hour of electricity in the United States is approximately 10 cents—slightly higher for residential customers and slightly lower for industrial customers. The health damages from coal alone are 3.2 cents/kWh, with some plants having external costs at 12 cents/kWh.

The book does not include the damages from climate change in its total costs, yet provides an intriguing calculation. If one assumed marginal damages to be $30/ton of carbon dioxide, the external costs from climate change per kilowatt hour are also in the neighborhood of 3 cents—doubling the estimated external costs.

Two other factors that will drive up the true external costs of energy are the impacts on ecosystems and the impacts on national security—either of which are extremely difficult to value. For health damages, the report uses the value of a statistical (human) life of US$6 million, which is based on a large universe of existing willingness-to-pay studies. With the dearth of such studies in the heterogeneous ecosystem context, one cannot credibly and comprehensively determine what the overall monetary damages on ecosystems are.

Even though a number of important effects have not been monetized, this report is the essential work in this area and belongs in the library of any serious researcher, policy maker, or writer working in this area. It is the most serious yet accessible assessment and literature review available of the external life-cycle costs of the energy system for the U.S. economy.

## Figures and Tables

**Figure:**